# The relationship between choroidal thickness and axis length and corneal curvature in high myopia

**DOI:** 10.12669/pjms.38.7.6409

**Published:** 2022

**Authors:** Hongwei Lu, Yongqing Guan, Guangchuan Wei, Na Li

**Affiliations:** 1 Hongwei Lu, Department of Ophthalmology, The Fourth Hospital of Hebei Medical University, Shijiazhuang 050000, Hebei Province, P.R. China; 2 Yongqing Guan, Department of Ophthalmology, The Fourth Hospital of Hebei Medical University, Shijiazhuang 050000, Hebei Province, P.R. China; 3 Guangchuan Wei, Department of Ophthalmology, The Fourth Hospital of Hebei Medical University, Shijiazhuang 050000, Hebei Province, P.R. China; 4 Na Li, Department of Ophthalmology, The Fourth Hospital of Hebei Medical University, Shijiazhuang 050000, Hebei Province, P.R. China

**Keywords:** Spectral-domain optical coherence tomography, Depth enhanced imaging technology, Choroidal thickness, Axial length

## Abstract

**Objectives:**

To measure choroidal thickness (CT) in highly myopia eyes using enhanced depth imaging spectral-domain optical tomography (EDI SD-OCT) and explore the relationship between the CT and axial length (AL) and corneal curvature.

**Methods:**

This study assessed 66 eyes of 33 patients with both eyes of high myopia (equivalent sphericity ≥-6D or AL≥26mm) who underwent treatment at the department of ophthalmology, fourth hospital of Hebei Medical University from August 2020 to August 2021(High-myopia group). The control group included 54 eyes from 27 healthy volunteers. The patients in the two groups were 18~40 years old. EDI SD-OCT was used to measure the CT of subfoveal choroidal thickness (SFCT) and CT from fovea to nasal and temporal sides at an interval of 500~2500μm. The intraocular pressure, diopter, ocular axis, corneal curvature, and CT were compared between the two groups. The correlation between CT, diopter and AL was measured by Pearson’s *r*.

**Results:**

The diopter, AL, vertical corneal curvature, and CT in the high-myopia group were significantly higher than those in the control group (*P<*0.05). There was significant correlation between SFCT and AL in both groups (*P<*0.05). There was a negative correlation between AL and CT in high-myopia group (r=-0.395, *P<*0.05), and a positive correlation between AL and CT in control group (r=0.29, *P<*0.05). There was a weak correlation between AL and gender, intraocular pressure, and horizontal corneal curvature(*P<*0.05), and a negative correlation between AL and diopter (r=-0.861, *P<*0.001).

**Conclusion:**

EDI SD-OCT can quantitatively measure the CT of high myopia. The CT of patients with high myopia was significantly thinner than that of the control group of the same age. There was a significant correlation between diopter, AL and CT, suggesting that AL is a parameter indicating the degree of myopia, and the change of CT may play a role in the occurrence and development of high myopia.

## INTRODUCTION

High myopia, also known as pathological myopia, refers to ametropia with diopter ≥ -6.0D or AL ≥26mm, accompanied by different degrees of degenerative changes of fundus retina and choroid.^1^ Moreover, high myopia can be accompanied by fundus pathological changes, such as choroidal neovascularization, chorioretinal atrophy, retinal detachment, macular hole and so on, seriously endangering vision and affecting normal life of patients.^2^

Choroidal dysfunction has been observed in many retina diseases, such as age-related maculopathy, myopic retinopathy, choroidal neovascular diseases, central serous retinopathy and Koyanagi Harada disease.^3^ Examination methods used to observe choroid have their own limitations. B-mode ultrasound can be used to observe the increase of CT, but it is not enough to provide cross-sectional image information. Indocyanine green angiography is used to observe the changes of choroidal vascular morphology and function, but it cannot provide data on the changes of choroidal morphology and thickness.^4^ Although the traditional coherent optical tomography can clearly display the structure of each layer of retina, it is unable to obtain a clear image of choroid due to the influence of factors such as choroidal blood flow, pigment and pigment epithelial layer scattering.^5^ Deep enhanced OCT technology, combined with eye tracking and image noise reduction technology, allows to obtain choroidal cross-sectional information in vivo.^6^ It provides a new method for the study of the pathogenesis of high myopia.

In the past, many studies have focused on the CT of high myopia and normal eyes. While the age of the patients varied, there were few biological data in the young population. In this study, we applied EDI-OCT technology to detect the distribution characteristics of CT in a group of high myopia, and further studied the AL, refraction correlation between corneal curvature and sub foveal CT.

## METHODS

All patients were from Shijiazhuang. The patients were informed and voluntarily participated in the study. The ethic committee of our hospital has approved this study (No. 2021KY328). Thirty three patients (66 eyes) with high myopia in both eyes diagnosed in the ophthalmology clinic of the fourth hospital of Hebei Medical University from August 2020 to August 2021 were selected the research objects, and 27 healthy volunteers (54 eyes) with normal binocular vision in the physical examination center of the hospital in the same period were selected as the normal control group.

### Inclusion Criteria:

- Age between 18 to 40 years old to ensure cooperation and more clear imaging to facilitate the measurement of CT.
- high-myopia group: Diopter ≥-6D or AL≥26mm, best corrected visual acuity ≥ 0.8; control group: diopter - 3.0~+ 1.0d, and the naked visual acuity ≥ 1.0;
- The intraocular pressure in the normal range of 10~21mmhg;

### Exclusion Criteria:

- Patients with > - 2.0D astigmatism
- Systemic diseases such as hypertension and diabetes.
- Diseases that affect choroidal structure, such as age-related macular degeneration(AMD), retinal detachment, macular neovascularization (CNV), retinal hole, retinal vein occlusion, etc.;
- Any choroidal or retinal detachment found by OCT without clear choroidal image;
- Patients with any ophthalmic diseases such as glaucoma, cataract, dominant strabismus and retinal diseases;
- Have received ophthalmic surgery and laser surgery.

This study has been registered with clinical trial center (No.: ChiCTR2100054670) International standard logarithmic visual acuity chart (GB11533-89); Slit lamp (SLM ophthalmic slit lamp microscope); Ophthalmoscope (YZ6E ophthalmoscope); computerized automatic Optometry (NIDEK ARK-510A); Non-contact tonometer (model: NT-2000); Non-contact biometric (Lenstar LS900); Optical coherence tomography Cirrus HD-OCT (model: 4000).

Patients in both groups were subjected to the choroidal thickness (CT) measurement using enhanced depth imaging spectral-domain optical tomography (EDI SD-OCT). All patients were routinely examined by international standard visual acuity chart, computer automatic optometry, slit lamp microscope, non-contact tonometer, direct fundus endoscopy and fundus photography.

General clinical data, including gender, age, eye type, intraocular pressure, naked visual acuity, diopter, corrected visual acuity, anterior segment and fundus were recorded for all patients. Choroidal thickness (CT) at the fovea of macula, 500μm, 1000μm, 1500μm, 2000μm and 2500μm on the temporal side, and 500μm, 1000μm, 1500μm, 2000μm, 2500μm on the nasal side was measured. To exclude the impact of the circadian rhythm changes in choroid on the test results, all examinations were performed between 8:00 and 16:00.^7^

AL measurement was performed using Lenstar LS900 non-contact biometrics. The patient was in a sitting position with the mandible on the jaw rack. After adjusting the eye position, the focal length was adjusted to measure. The biometric values of eye axis, horizontal corneal curvature and vertical corneal curvature of all observation subjects were measured three times. The data were analyzed and read with the analysis software, and the average value was taken.

Detection of choroid was done using Cirrus HD-OCT depth enhanced imaging technology (Zeiss, Germany). EDI mode was used to scan the macular region of the posterior pole at 0° with a scanning line with a length of 6mm. SFCT and CT from fovea to nasal and temporal sides were measured every 500μm to 2500μm. Each eye was scanned three times and the average of the three measurements was taken as the final research data. Scanned images were stored in the computer.

### Statistical Analysis

SPSS 22.0 statistical software was used for data processing. The measurement data is expressed in (*X̄*±*s*), and the independent sample t-test is conducted. Pearson correlation was used to analyze the relationship between various factors and CT in each group. The confidence interval was 5~95%; *P<*0.05 was considered statistically significant.

## RESULTS

A total of 66 eyes of 33 patients (9 males, 24 females) with high myopia were selected as high-myopia group, the average age was (27.55±4.66) years. The control group included 54 eyes of 27 healthy volunteers, seven males, 20 females, the average age was (27.33±5.65) years. There was no significant difference in gender and age between the groups (*P>*0.05).

There was no significant difference in intraocular pressure and horizontal corneal curvature between the two groups (*P>*0.05), but the diopter, AL and vertical corneal curvature in the high-myopia group were significantly higher than those in the control group (*P<*0.05) (Table-I).

**Table-I:** Intraocular pressure, diopter, AL and corneal curvature were compared between the two groups (*X̅*±*S*).

Group	Intraocular pressure(mmhg)	Diopter(D)	AL(mm)	Horizontal corneal curvature(D)	Vertical corneal curvature(D)
High-myopia group	14.55±2.25	-8.12±1.95	26.50±0.15	43.24±0.20	44.59±0.21
Control group	14.71±2.22	-1.44±1.19	23.86±0.11	43.20±0.14	43.99±0.14
t	0.378	-13.72	-13.72	-0.108	-2.33
P	0.706	<0.001	<0.001	0.851	0.021

**Fig.1:**
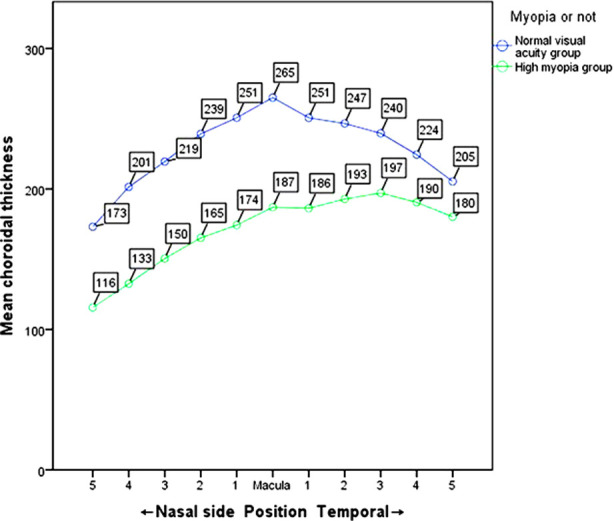
The distribution of CT in the two groups. In the high-myopia group, the thickest choroid is in the temporal side, the next in the fovea, and the thinnest in the nasal side; Control group: the choroid is the thickest in the fovea, the temporal side is the second, and the nasal side is the thinnest.

SFCT in the high-myopia group was significantly thinner than in the control group, and the CT at each measurement site decreased significantly (*P<*0.001). In the high-myopia group, the temporal choroid was the thickest (197μm), followed by the fovea (187μm), and the nasal choroid was the thinnest (116μm). In the control group, the fovea choroid was the thickest (265μm), followed by the temporal choroid (205μm), and the nasal choroid was the thinnest (173μm). The average CT of each measurement site in the two groups is listed in Table-II (Fig.1).

**Table-II:** CT at different distances from macular fovea in each quadrant of the two groups (μm,(*X̅*±*S*))

Position	High-myopia group	Control group	F	t	P
Number of eyes	66	54			
SFCT	186.94±54.337	265.00±65.641	0.07	7.63	<0.001
Nasal side 2500μm	115.61±41.216	173.07±51.540	1.868	6.38	<0.001
Nasal side 2000μm	132.52±53.437	201.44±54.969	0.526	6.89	<0.001
Nasal side 1500μm	150.47±55.354	219.46±55.714	0.316	6.73	<0.001
Nasal side 1000μm	165.14±56.825	239.22±56.087	0.014	7.09	<0.001
Nasal side 500μm	174.3±57.553	250.65±52.778	0.706	7.45	<0.001
Temporal 2500μm	180.14±55.536	205.31±49.389	0.596	2.58	0.01
Temporal 2000μm	190.42±53.635	224.43±49.624	0.249	3.55	<0.001
Temporal 1500μm	197.12±54.916	239.61±49.686	0.540	4.37	<0.001
Temporal 1000μm	192.84±55.182	246.61±49.349	0.762	5.53	<0.001
Temporal 500μm	186.20±51.933	250.63±49.007	1.294	6.88	<0.001

**Fig.2:**
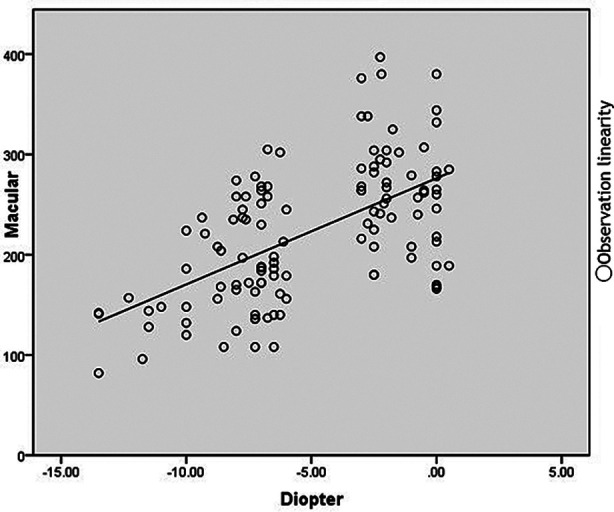
Scatter plot shows the relationship between CT and diopter in macular area, and CT is positively correlated with diopter.

There was no correlation between the intraocular pressure and corneal curvature (*P>*0.05), and a significant correlation between SFCT and AL in both groups (*P<*0.05). In high-myopia group, temporal CT 2500μm, 2000μm and 1500μm had no correlation with diopter and AL(*P>*0.05). CT in other positions had a significant correlation with diopter and AL (*P<*0.05). In the control group, nasal CT 2500μm, 2000μm, 1500μm, 1000μm and 500μm had no significant correlation with diopter and AL(*P>*0.05). SFCT and temporal 2500μm had no significant correlation with diopter (*P>*0.05) but had significant correlation with AL (*P<*0.05). CT in other positions significantly correlated with diopter and AL (*P<*0.05). (Table-III and Table-IV) (Fig.2).

**Table-III:** Relationship between CT, diopter and AL in high-myopia group.

Position		Diopter	AL
SFCT	r	0.396	-0.395
	P	0.001	0.001
Temporal 2500μm	r	0.076	-0.083
	P	0.549	0.512
Temporal 2000μm	r	0.113	-0.183
	P	0.375	0.149
Temporal 1500μm	r	0.229	-0.281
	P	0.068	0.025
Temporal 1000μm	r	0.311	-0.374
	P	0.021	0.002
Temporal 500μm	r	0.328	-0.406
	P	0.008	0.001
Nasal side 500μm	r	0.419	-0.453
	P	0.001	<0.001
Nasal side 1000μm	r	0.445	-0.451
	P	<0.001	<0.001
Nasal side 1500μm	r	0.457	-0.420
	P	<0.001	0.001
Nasal side 2000μm	r	0.457	-0.375
	P	<0.001	0.002
Nasal side 2500μm	r	0.428	-0.354
	P	<0.001	0.004

**Table-IV:** Relationship between CT, diopter and AL in control group.

Position		Diopter	AL
SFCT	r	-0.207	0.290
	P	0.132	0.034
Temporal 2500μm	r	-0.269	0.397
	P	0.049	0.003
Temporal 2000μm	r	-0.351	0.434
	P	0.009	0.001
Temporal 1500μm	r	-0.304	0.432
	P	0.025	0.001
Temporal 1000μm	r	-0.291	0.402
	P	0.033	0.003
Temporal 500μm	r	-0.222	0.347
	P	0.107	0.01
Nasal side 500μm	r	-0.03	0.138
	P	0.829	0.319
Nasal side 1000μm	r	0.013	0.111
	P	0.926	0.425
Nasal side 1500μm	r	0.147	-0.011
	P	0.289	0.938
Nasal side 2000μm	r	0.099	0.021
	P	0.476	0.88
Nasal side 2500μm	r	0.180	0.018
	P	0.192	0.9

**Fig.3:**
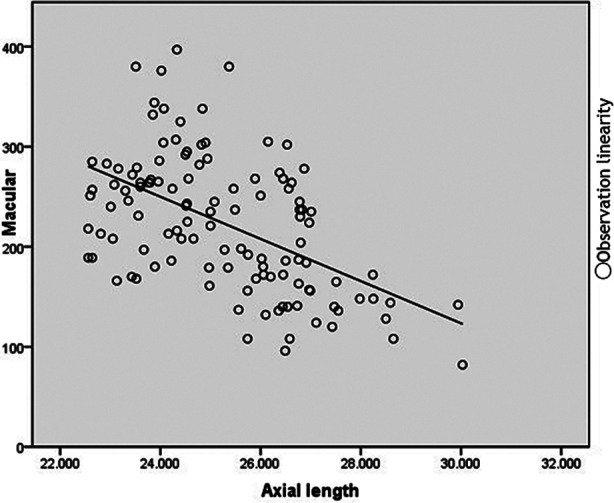
Scatter plot shows the relationship between CT and AL in macular area, and CT is negatively correlated with AL.

There was no correlation between AL and age, left or right eye, vertical corneal curvature (*P>*0.05), but there was a weak correlation with gender, intraocular pressure and horizontal corneal curvature (*P<*0.05), and there was a negative correlation between AL and diopter (r=-0.861, *P<*0.001) (Fig.3).

## DISCUSSION

The results of our study show that high myopia patients have markedly lower CT compared to control group of the same age. There is a significant difference in the CT under the macular fovea, and a significant correlation between diopter, AL and CT. Changes of choroidal outcome and function are closely related to the occurrence and development of myopia. Therefore, the study of the subtle morphological results and functional changes of choroid may help to reveal the etiology of high myopia and monitor the development of related fundus pathological changes.^8^

Our study showed that there was no correlation between the CT and age. The selected observation population was in the 18~40 year’s old range, with a small age span which reduced the impact of age factors on CT. The CT in the high-myopia group was significantly thinner than that in the control group, which was consistent with previous studies.^9^

To study the characteristics of CT distribution in macular area, we measured 11 points respectively. The results showed that in high-myopia group, the thickest choroid was in the temporal side, followed by the fovea, and the nasal side being the thinnest. In the control group, the choroid was the thickest in the fovea, followed by the temporal side, and the nasal side was the thinnest. We showed that the nasal quadrant was thinner than the macular and temporal quadrant, which is consistent with previous reports.^10^ This phenomenon may be caused by choroidal watershed and fetal choroidal fissure (which closes the lower choroid at seven weeks).^11^ The watershed area in the macular fovea may lead to the thinning of the choroid during the expansion of the eyeball.^12^

Read et al.^9^ showed that CT significantly correlated with diopter and age. When diopter increased by Minus one D, SFCT was reduced by 19μm, and the average SFCT of myopia was 16% thinner than that of emmetropia. Gupta et al.^13^ found that in young patients with high myopia, CT was significantly thinner than that of normal eyes of the same age, AL, intraocular pressure. The presence of posterior staphyloma and chorioretinal atrophy significantly affect CT, Jin et al.^14^ suggested that CT in Chinese children was closely related to AL and diopter. Fujiwara et al.^15^ first pointed out that SFCT in high myopia was positively correlated with diopter. That is, SFCT decreased by 8.7μm for every Minus one D increase in diopter. Vincent et al. 16 found that there was no significant difference in retinal thickness between the eyes with large myopia diopter and contralateral eyes, but CT decreased significantly. Ho et al.^17^ found that SFCT decreased by 6.205μm for every Minus one D increase in diopter. Chen et al.^18^ concluded that in general, there is no significant difference in SFCT between left and right eyes, which is in agreement with the results of our study.

The AL of the high-myopia group in our study was significantly higher than that of the control group. Our results are consistent with the results of previous studies. Teberik et al.^19^ showed that there was a negative correlation between CT and AL in adult patients with high myopia (r=-0.490, *P<*0.05). Flores mores et al.^20^ found that CT decreased by 25.91μm for every 1mm increase in AL. These findings were also confirmed by Takahashi et al.^21^

### Limitations

1) CT was measured manually with errors. 2) In this study, only a certain range of CT in the fovea, temporal and nasal sides were measured, but the upper and lower CT were not involved. 3) The sample size of this study is small, and patients come from the same city. More studies are needed to investigate these limitations when analyzing CT in high myopia in the future.

## CONCLUSION

EDI SD-OCT can quantitatively measure the CT of high myopia. The CT of adult patients with high myopia was significantly thinner than that of the control group of the same age. There is a significant correlation between diopter, AL and CT, suggesting that AL is a parameter indicating the degree of myopia, and the change of CT may play a role in the occurrence and development of high myopia.

### Authors’ contributions:

**HL** conceived and designed the study.

**YG, GW and NL** collected the data and performed the analysis.

**HL** was involved in the writing of the manuscript and the integrity of the study.

All authors have read and approved the final manuscript.

## References

[1] Morgan IG, Ohno-Matsui K, Saw SM (2012). Myopia. Lancet.

[2] Read SA, Fuss JA, Vincent SJ, Collins MJ, Alonso-Caneiro D (2019). Choroidal changes in human myopia:insights from optical coherence tomography imaging. Clin Exp Optom.

[3] Shehzad M, Aziz T (2014). Choroidal neo-vascularization presentation in younger age group (pre-senile). J Pak Med Assoc.

[4] Teussink MM, Breukink MB, van Grinsven MJJP, Hoyng CB, Klevering BJ, Boon CJF (2015). OCT Angiography Compared to Fluorescein and Indocyanine Green Angiography in Chronic Central Serous Chorioretinopathy. Invest Ophthalmol Vis Sci.

[5] Theelen T, Teussink MM (2018). Inspection of the Human Retina by Optical Coherence Tomography. Methods Mol Biol.

[6] Hagag AM, Mitsios A, Gill JS, Nunez Do, Rio JM, Theofylaktopoulos V, Houston S (2020). Characterisation of microvascular abnormalities using OCT angiography in patients with biallelic variants in USH2A and MYO7A. Br J Ophthalmol.

[7] Quintela T, Furtado A, Duarte AC, Goncalves I, Myung J, Santos CRA (2021). The role of circadian rhythm in choroid plexus functions. Prog Neurobiol.

[8] Tian J, Marziliano P, Baskaran M, Tun TA, Aung T (2012). Automatic measurements of choroidal thickness in EDI-OCT images. Annu Int Conf IEEE Eng Med Biol Soc.

[9] Read SA, Collins MJ, Vincent SJ, Alonso-Caneiro D (2013). Choroidal thickness in myopic and nonmyopic children assessed with enhanced depth imaging optical coherence tomography. Invest Ophthalmol Vis Sci.

[10] Zha Y, Zhuang J, Feng W, Zheng H, Cai J (2020). Evaluation of choroidal thickness in amblyopia using optical coherence tomography. Eur J Ophthalmol.

[11] Centini G, Imperatore A, Morelli M, Rosignoli L, Passamonti U, Caprioli F (2013). Bifid choroid plexus:always a normal fetal brain structure variant?. Congenit Anom (Kyoto).

[12] Lutty GA, McLeod DS (2018). Development of the hyaloid, choroidal and retinal vasculatures in the fetal human eye. Prog Retin Eye Res.

[13] Gupta P, Cheung CY, Saw SM, Bhargava M, Tan CS, Tan M (2015). Peripapillary choroidal thickness in young Asians with high myopia. Invest Ophthalmol Vis Sci.

[14] Jin P, Zou H, Zhu J, Xu X, Jin J, Chang TC (2016). Choroidal and Retinal Thickness in Children with Different Refractive Status Measured by Swept-Source Optical Coherence Tomography. Am J Ophthalmol.

[15] Fujiwara T, Imamura Y, Margolis R, Slakter JS, Spaide RF (2009). Enhanced depth imaging optical coherence tomography of the choroid in highly myopic eyes. Am J Ophthalmol.

[16] Vincent SJ, Collins MJ, Read SA, Carney LG (2013). Retinal and choroidal thickness in myopic anisometropia. Invest Ophthalmol Vis Sci.

[17] Ho M, Liu DTL, Chan VCK, Lam DSC (2013). Choroidal thickness measurement in myopic eyes by enhanced depth optical coherence tomography. Ophthalmology.

[18] Chen FK, Yeoh J, Rahman W, Patel PJ, Tufail A, Da Cruz L (2012). Topographic variation and interocular symmetry of macular choroidal thickness using enhanced depth imaging optical coherence tomography. Invest Ophthalmol Vis Sci.

[19] Teberik K, Kaya M (2017). Retinal and Choroidal Thickness in Patients with High Myopia without Maculopathy. Pak J Med Sci.

[20] Flores-Moreno I, Lugo F, Duker JS, Ruiz-Moreno JM (2013). The relationship between axial length and choroidal thickness in eyes with high myopia. Am J Ophthalmol.

[21] Takahashi A, Ito Y, Iguchi Y, Yasuma TR, Ishikawa K, Terasaki H (2012). Axial length increases and related changes in highly myopic normal eyes with myopic complications in fellow eyes. Retina.

